# Transgene Silencing and Transgene-Derived siRNA Production in Tobacco Plants Homozygous for an Introduced *AtMYB90* Construct

**DOI:** 10.1371/journal.pone.0030141

**Published:** 2012-02-17

**Authors:** Jeff Velten, Cahid Cakir, Eunseog Youn, Junping Chen, Christopher I. Cazzonelli

**Affiliations:** 1 United States Department of Agriculture - Agricultural Research Service, Lubbock, Texas, United States of America; 2 Department of Computer Science, Texas Tech University, Lubbock, Texas, United States of America; 3 Australian Research Council - Centre of Excellence in Plant Energy Biology, Research School of Biology, Australian National University, Canberra, Australian Capital Territory, Australia; German Cancer Research Center, Germany

## Abstract

Transgenic tobacco (*Nicotiana tabacum*) lines were engineered to ectopically over-express *AtMYB90* (*PAP2*), an R2–R3 Myb gene associated with regulation of anthocyanin production in *Arabidopsis thaliana*. Independently transformed transgenic lines, Myb27 and Myb237, accumulated large quantities of anthocyanin, generating a dark purple phenotype in nearly all tissues. After self-fertilization, some progeny of the Myb27 line displayed an unexpected pigmentation pattern, with most leaves displaying large sectors of dramatically reduced anthocyanin production. The green-sectored 27Hmo plants were all found to be homozygous for the transgene and, despite a doubled transgene dosage, to have reduced levels of *AtMYB90* mRNA. The observed reduction in anthocyanin pigmentation and *AtMYB90* mRNA was phenotypically identical to the patterns seen in leaves systemically silenced for the *AtMYB90* transgene, and was associated with the presence of *AtMYB90*-derived siRNA homologous to both strands of a portion of the *AtMYB90* transcribed region. Activation of transgene silencing in the Myb27 line was triggered when the 35S::*AtMYB90* transgene dosage was doubled, in both Myb27 homozygotes, and in plants containing one copy of each of the independently segregating Myb27 and Myb237 transgene loci. Mapping of sequenced siRNA molecules to the Myb27 TDNA (including flanking tobacco sequences) indicated that the 3′ half of the *AtMYB90* transcript is the primary target for siRNA associated silencing in both homozygous Myb27 plants and in systemically silenced tissues. The transgene within the Myb27 line was found to consist of a single, fully intact, copy of the *AtMYB90* construct. Silencing appears to initiate in response to elevated levels of transgene mRNA (or an aberrant product thereof) present within a subset of leaf cells, followed by spread of the resulting small RNA to adjacent leaf tissues and subsequent amplification of siRNA production.

## Introduction

Dramatic variability of transgene expression, including complete silencing of the introduced gene or genes, has been a factor impacting the success of plant genetic engineering since its inception. The observed variability in expression levels of what appear to be identical transgene constructs has been linked to multiple molecular factors such as high transcription levels, alterations to the copy number and orientation of introduced DNA, and the characteristics of closely linked plant genetic material [1], [2], [3], [4], [5], [6]. Co-suppression of unlinked homologous plant genes is often associated with transgene silencing and represents one of the first published observations of RNA-based gene regulation [7], [8], [9]. Silencing of introduced transgenes is frequently attributed to post-transcriptional gene silencing (PTGS), one of many small RNA (smRNA) based molecular processes occurring in plants. Short, 21–24 nucleotide (nt), RNA molecules are increasingly implicated as important regulators of critical biological processes, including; tissue development, pathogen defense, stress response, and epigenetic gene silencing in plants (for recent reviews see [10], [11], [12], [13], [14], [15], [16], [17], [18]). Most of the regulatory activities associated with these smRNAs appear to involve direct alterations togene activity, impacting mRNA production, message stability, and/or translation.

The ‘gold standard’ for inducing gene silencing in plants involves the production of double-stranded RNA (dsRNA), usually from genetic constructs engineered to produce self-complementary hairpin RNA transcripts [19], [20], [21]. The initiation of naturally occurring transgene silencing is also generally believed to involve the production of some form of double-stranded RNA. However, despite dramatic advances in our understanding of the molecular and biochemical components of plant gene silencing, exactly how and why it is initiated often remains unclear. Transgenes that have been rearranged or duplicated during integration into the host genome appear to be prone to silencing, possibly due to the direct production of complementary RNA. In systems involving virus-induced gene silencing (VIGS), it is thought that replicative intermediates, and viral RNA secondary structures, induce the production of small interfering RNA (siRNA), a process that may be amplified by RNA-dependent RNA polymerase (RdRP) activity to enhance disruption of virus replication and spread [6], [22]. It becomes less clear what factor(s) trigger the initiation of silencing with transgenes that lack evidence of abnormal DNA structures or unusual genome locations. It is generally assumed that in these cases, silencing starts in response to the presence of aberrant RNA (e.g. transcripts or RNA fragments lacking 5′ cap structures or 3′ poly-A tails [1], [23]) and that when the amount of abnormal RNA reaches a critical level, silencing targeting homologous mRNA is initiated.

Due to the stochastic nature of most transgene silencing it has proved difficult to directly address the nature of molecular factors associated with the initiation, spread and maintenance of transgene-targeted silencing. There have been a few reports where transgene silencing was found to start in response to the doubling of transgene dosage occurring within plants made homozygous for a single copy transgene [24], [25], [26], [27], [28], [29], [30], [31], [32]. These examples provide experimental systems that are theoretically amenable to a molecular examination of early silencing processes, but are often complicated by inconsistent or incomplete transgene silencing. We have developed a system [33] where silencing of an introduced *Arabidopsis* myb transcription factor is consistently and reproducibly associated with doubling of the transgene copy number that occurs within homozygotes (over at least 3 generations). The structurally intact, single copy, 35S::*AtMYB90* transgene provides a simple visual indicator of silencing (reduced anthocyanin production [34], [35]) and does not introduce an additional copy of any native tobacco gene or genes (e.g. [36], [37], [38]). The lack of an endogenous copy of the transgene reduces the potential for pleotropic effects, and makes it easier to identify the origin of any smRNA targeting the transgene. In this report we describe the *AtMYB90* system, gene-dosage induced transgene silencing, and the nature of the small RNA correlated with silencing of the introduced myb transgene.

## Results

### A 35S::*AtMYB90* transgenic tobacco line, Myb27, displays transgene silencing in plants homozygous, but not hemizygous, for the transgene locus

**Figure 1:**
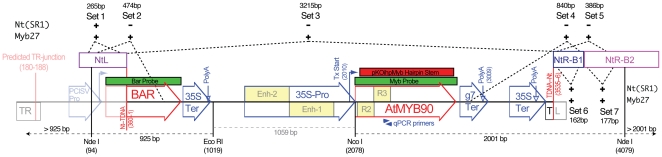
Graphic map of TDNA containing the 35S::*AtMYB90* construct. The main map shown represents the TDNA construct (containing a 35S::*AtMYB90* transgene) used to transform *N. tabacum*. Flagged vertical lines indicate TDNA/plant DNA junctions cloned from the Myb27 line, with plant segments shown above the main map (**NtL**, tobacco sequence at the TR border; **NtR-B1 and NtR-B2**, tobacco sequences at the TL border). Segments of the original plasmid TDNA that are absent at the Myb27 locus are shown as a faded graphic. Labels include: **TR** (right TDNA border); **PClSV-Pro** (Peanut Chlorotic Streak Virus promoter); **BAR** (basta resistance coding region); **35S-Ter** (CaMV 35S transcription termination signal); **35S-Pro** (CaMV 35S promoter with duplicated enhancer region [**Enh-1**, **Enh-2**]); ***AtMYB90*** (*AtMYB90* coding region [the Myb **R2–R3** repeats are indicated]); **g7-Ter** (transcription termination signal from the gene-7 of octopine TDNA); **TL** (left TDNA border). The locations of primers used for qRTPCR are indicated by blue arrowheads and the segment of the *AtMYB90* coding region used in a hairpin expression vector (pKOihpMyb) is indicated by a red box. Dashed lines show the boundaries of PCR amplimers used to confirm the structure of the T-DNA insert (“**+**” indicates predicted PCR product observed, “**−**” indicates no product of the predicted size; primer sequences are listed in Additional files, Table S1). The PCR templates used were total plant leaf DNA from the **Myb27** line and from untransformed tobacco, **Nt (SR1)**. The BAR and Myb probes used for blot hybridization are indicated by green boxes above the map and restriction enzyme cleavage sites used for Southern blot analysis, including resulting fragment sizes, are shown below the main map.

**Figure 2:**
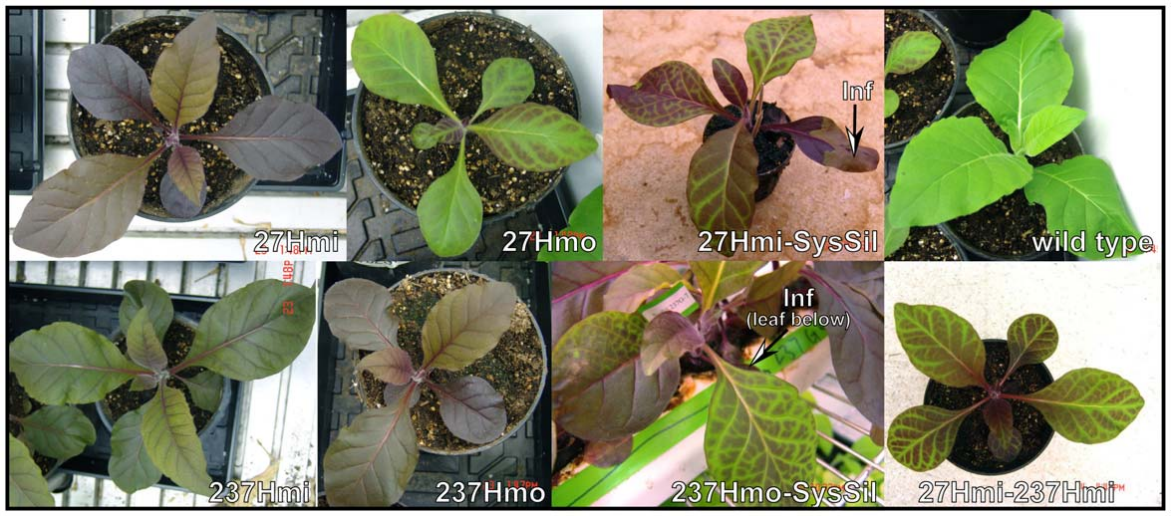
Plant pigmentation phenotypes. Phenotypes of 35S::*AtMyb90* transgenic lines Myb27 and Myb237 that are hemizygous (27Hmi, 237Hmi) or homozygous (27Hmo, 237Hmo) for the transgene. Also shown are 27Hmi and 237Hmo plants displaying systemic silencing (27Hmi-SysSil, 237Hmo-SysSil) induced by pKOhpMyb-*Agrobacterium* infiltration (see hairpin stem, Fig. 1) of older leaves (infiltration sites indicated by ‘**Inf**’ arrows). A similar pattern of silencing is seen in the offspring from a 27Hmo×237Hmo cross (the plant shown is hemizygous for both the Myb27 and Myb237 transgene loci [27Hmi–237Hmi]).

**Figure 3:**
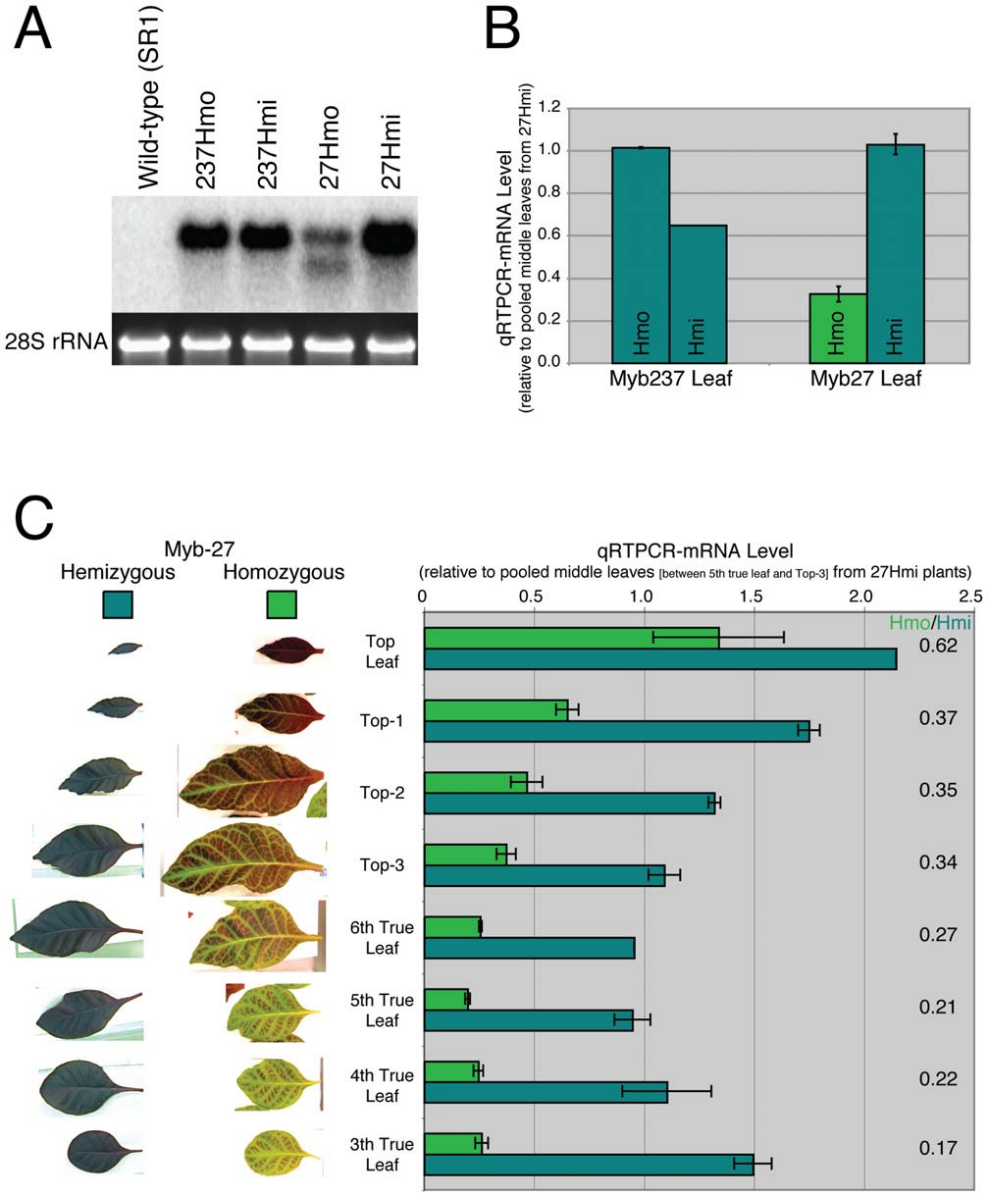
35S::*AtMyb90* transcription in Myb27 and Myb237 lines. A. Northern blot autoradiogram of total RNA from 35S::*AtMyb90* transgenic plants. The *AtMYB90* coding region was used as probe (see Fig. 1) against equal RNA loadings (confirmed by 28S rRNA fluorescence, bottom row) from 27Hmi and 237Hmo leaf material (leaves used were similar to the 5th True Leaf pictured in 3C). The 27Hmo blot shows two bands of approximately 1050 nt and 600 nt. B. Comparison of mRNA levels (qRTPCR) using leaf tissue from 27Hmo (partially-green), 27Hmi (purple), 237Hmo (purple) and 237Hmi (purple) plants. The individual samples were normalized to total RNA from a pooled leaf collection of 27Hmi leaves (5th true leaf through the third from the top were combined). Error bars represent standard error for 2–3 biological replicates. C. AtMYB90 mRNA quantification (qRTPCR) from 27Hmo and 27Hmi matched leaves of varying ages (shown at the left). In order to match leaf development between plants sampling was limited to the oldest (the 3rd, 4th, 5th and 6th true leaves) and youngest (top leaf, Top-1, Top-2, Top-3) leaves. Error bars represent standard error for 2–3 biological replicates. The ratio of homozygous/hemizygous values for each pair of matched leaves is indicated at the right. Individual samples were normalized to the same pooled 27Hmi leaf RNA used for part B.

A gene construct engineered to express an *Arabidopsis* R2R3-Myb transcription factor associated with regulation of anthocyanin pigment production (*AtMYB90* or *PAP2*) [35] was introduced into *Nicotiana tabacum* (Petite Havana SR1) by *Agrobacterium tumifaciens* transformation (Fig. 1). Several of the resulting independently transformed transgenic lines demonstrated ectopic over-expression of *AtMYB90* by producing a darkly pigmented (anthocyanins) phenotype in nearly all plant tissues (Fig. 2, 27Hmi and 237Hmi) [33]. When selfed, most of these plants, including the Myb237 line, produced the expected 3∶1 ratio of pigmented (purple) to wild type (green) offspring, consistent with a single dominant transgene locus. However, one purple line, Myb27, consistently displayed an approximately 2∶1∶1 ratio of purple to partially-pigmented to fully-green phenotypes in its R_1_ offspring. Following out-crossing of the R_1_ progeny to wild type tobacco, the partially pigmented Myb27 plants were all found to be homozygous for the introduced *AtMYB90* transgene (27Hmo). All 27Hmo plants were found to reproducibly produce reduced anthocyanin levels relative to their hemizygous (27Hmi) parent or siblings (Fig. 2). In 27Hmo leaves, areas of near wild type green pigmentation developed during the expansion and maturation of leaves that emerged from the apical meristem displaying a fully purple phenotype (Fig. 2 and Fig. 3C). The reduced anthocyanin content seen in expanding 27Hmo leaves is most pronounced close to leaf veins, spreading into inter-vein tissue as the leaf expands. This pattern of pigment loss is very similar to that seen in purple plants (27Hmi, 237Hmi, 237Hmo, Fig. 2) in which systemic silencing (SysSil) has been induced by infiltration of older leaves with an *A. tumefaciens* strain containing a hairpin construct (pKOihpMyb) that produces dsRNA homologous to the *AtMYB90* coding region (indicated in Fig. 1). The similarity between the 27Hmo and SysSil phenotypes (Fig. 2, 27 Hmo, 27Hmi-SysSil and 237Hmo-SysSil) suggested that gene silencing contributes to the observed pattern of pigment reduction seen in 27Hmo plants.

Silencing of the Myb transgene can also function in trans as demonstrated by a 27Hmo-like pattern of anthocyanin reduction in the offspring of 27Hmo×237Hmo crosses (27Hmi–237Hmi in Fig. 2). The resulting offspring are hemizygous for both the Myb27 and Myb237 TDNA loci, genotypes that separately display a fully pigmented purple phenotype (Fig. 2). Offspring resulting from self fertilization of the 27Hmi–237Hmi plants displayed a mixture of pigmentation patterns consistent with independent segregation of the Myb27 and Myb237 transgene loci. The observed transgene silencing seen in 27Hmi–237Hmi plants indicates that 27Hmo silencing is not due to disruption of a native tobacco gene at the Myb27 TDNA insertion site. The silenced 27Hmi–237Hmi plants contain a single, wild type, copy of any tobacco genes that might have been disrupted by Myb27 or Myb237 TDNA insertion, making them genetically the same as fully purple 27Hmi or 237Hmi plants at the TDNA loci.

### Reduced anthocyanin pigmentation in 27Hmo leaves correlates with decreased steady-state *AtMYB90* mRNA levels

Leaf total RNA samples from 27Hmi, 27Hmo, 237Hmi and 237Hmo leaves were analyzed using northern blot hybridization. The silenced 27Hmo sample contained reduced *AtMYB90* mRNA levels and displayed an altered pattern of hybridizing RNA bands when compared to samples from fully pigmented 27Hmi, 237Hmo and 237Hmi leaves (Fig. 3A). The presence of a shorter *AtMYB90*-hybridizing RNA band in the 27Hmo sample (Fig. 3A) is likely the result of small RNA-associated cleavage of mature *AtMYB90* mRNA. The intact *AtMYB90* transcripts seen in the 27Hmo sample most likely represent mRNA from the unsilenced, purple tissues that remain in the sampled leaf tissues (see representative leaves, Fig. 3C).

Quantitative reverse transcription polymerase chain reaction (qRTPCR) confirmed the expected gene-dosage associated increase in *AtMYB90* message in 237Hmo leaves relative to 237Hmi material. However, consistent with the northern data, Myb27 plants displayed an inverse correlation between transgene dosage and message levels (Fig. 3B). The reduction in anthocyanin pigmentation observed in maturing 27Hmo leaves correlated well with steady state *AtMYB90* mRNA levels (Fig. 3C). The relative *AtMYB90* message levels in Myb27 leaves varied with leaf age in both 27Hmi and 27Hmo plants, but were consistently lower in 27Hmo material when compared to age-matched 27Hmi leaves (Fig. 3C). Except when indicated, middle leaves (5^th^–9th true leaves) were used for subsequent mRNA and siRNA analysis.

Sequencing of PCR amplimers from the ends of *AtMYB90* cDNA (generated by rapid amplification of cDNA ends [RACE]) identified the same transcription start and stop points for mRNA isolated from both silenced (27Hmo) and unsilenced (27Hmi, 237Hmo) plants (the transcribed region is indicated on Fig. 1 and in Additional file - Fig. S1). The invitrogen™ GeneRacer™ kit and protocols used to identify the 5′ end of mature 35S::*AtMYB90* transcripts were designed to specifically amplify capped 5′ mRNA ends.

In summary, the primary *AtMYB90* transcript produced in Myb27 plants is structurally normal and mRNA levels in leaf tissues correlate well with anthocyanin pigment production and accumulation.

### Silencing of the 35S::*AtMYB90* transgene in 27Hmo plants is reversible

Excised green tissue segments from silenced 27Hmo leaves were surface sterilized and induced to form callus on artificial culture media. Resulting de-differentiated tobacco cells displayed strong anthocyanin production, indicating a reversal of transgene silencing (Additional file - Fig. S2). The observed reversal of silencing in propagating cells is similar to that seen previously with a silenced green fluorescent protein (GFP) transgene in *Nicotiana benthamiana* [26], [39].

### Genomic DNA organization and structure at the Myb27 TDNA insertion locus

**Figure 4:**
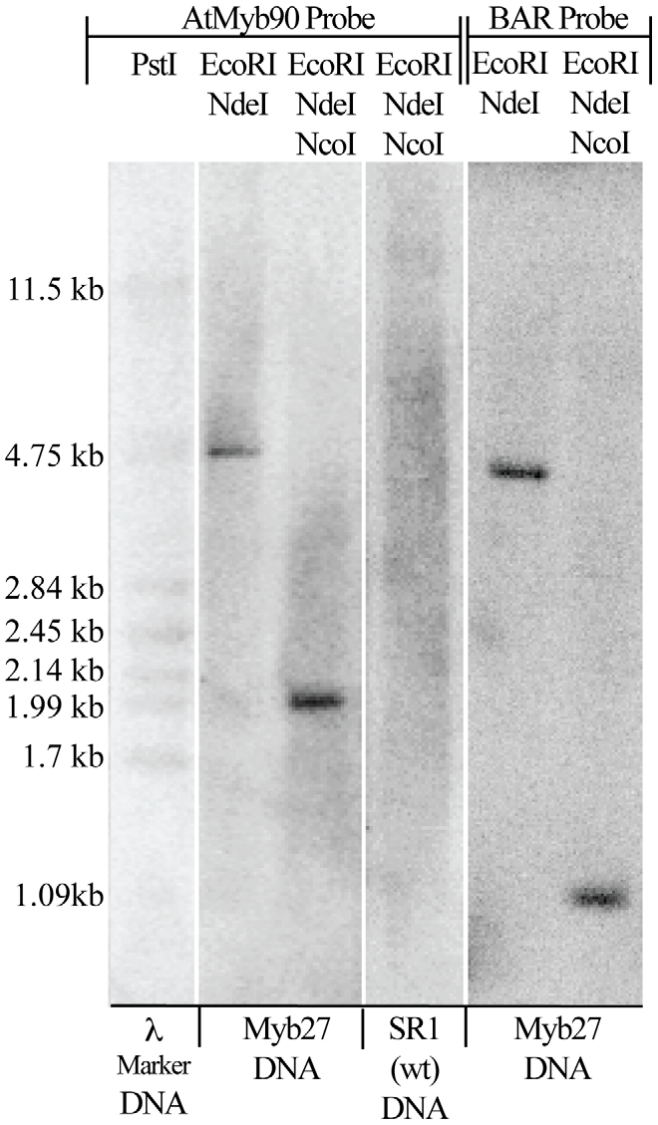
Southern Blot Analysis of Myb27 TDNA Insert. 27Hmo DNA digested with EcoRI plus NcoI, or EcoRI plus NcoI plus NdeI (see Fig. 1) were electrophoretically separated, blotted and probed with either BAR or *AtMyb90* coding regions. The single large EcoRI+NcoI bands (BAR, ∼4.5 kb and *AtMyb90*, ∼5 kb) that were shortened by NdeI digestion to a smaller band for each probe (BAR, 925 bp and Myb, 2001 bp).

The TDNA-plant junction sequences were cloned using thermal asymmetric interlaced polymerase chain reaction (TAIL-PCR [40]) and adaptor-primed PCR (GenomeWalker™) applied to total-DNA extracted from 27Hmo plants. Sequencing of the resulting Myb27 junction clones provided 360 bp of tobacco genomic DNA sequence flanking the TDNA right border (TR) (the tobacco sequence is indicated as NtL in Fig. 1 to maintain a left-to-right orientation for the 35S::*AtMYB90* transgene) and 793 bp adjacent to the TDNA left border (TL) (flanking sequence is indicated as NtR-B1 and NtR-B2 in Fig. 1). The genomic context of the Myb27 transgene locus was confirmed by demonstrating that PCR amplification using primers spanning the cloned plant-TDNA boundaries (e.g. set 4, Fig. 1) was limited to DNA from the Myb27 line (Fig. S3-B). The cloned NtL junction sequence confirmed previous PCR data indicating that the TR border, and part of the BAR selectable marker, were missing in the Myb27 line (Fig. 1 [33]). Long PCR using primers specific to the two cloned Myb27 TDNA-tobacco junctions (set 3, Fig. 1) amplified a ∼3215 bp fragment that was exclusive to the Myb27 line (Fig. S3-A). End-to-end sequencing of this PCR product confirmed the structure of the TDNA insert present within the Myb27 line. Southern blot hybridization was used to confirm that the Myb27 line contains a single copy of the *AtMYB90* transgene. Digested 27Hmo DNA (EcoRI plus NcoI, or EcoRI plus NcoI plus NdeI, see Fig. 1) were electrophoretically separated, blotted and probed with either BAR or *AtMyb90* coding regions. Each probe hybridized to a single large EcoRI+NcoI band (BAR, ∼4.5 kb and *AtMyb90*, ∼5 kb) that were shortened by NdeI digestion to one smaller band for each probe (Fig. 4). NdeI cuts only within the flanking tobacco DNA at both ends of the Myb27 TDNA insert [see Fig. 1], releasing smaller DNA bands of the sizes predicted for the single TDNA insert displayed in Fig. 1 (BAR, 925 bp and Myb, 2001 bp).

**Figure 5:**
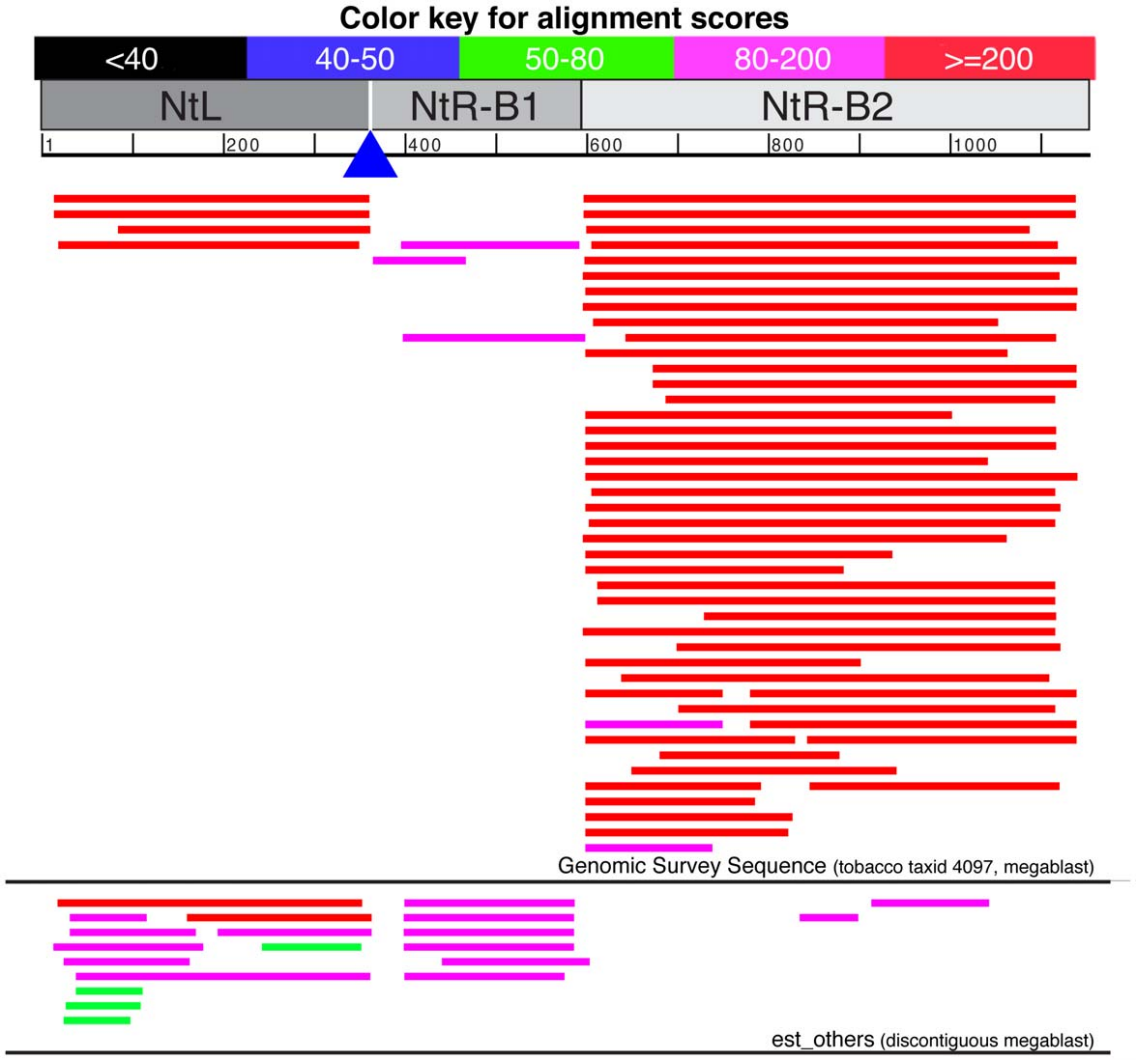
Graphic representation of GenBank matches to cloned tobacco flanking sequences in Myb27. The top horizontal bar represents the color scale for NCBI/BLAST alignment scores represented by the alignment bars shown below. The NtL, NtR-B1 and NtR-B2 sequencers were combined into a single string prior to BLAST alignment (the blue triangle indicates the T-DNA insertion site). The combined tobacco junction sequences were BLAST aligned [discontiguous megablast] against tobacco genomic sequences [Genomic Survey Sequence - tobacco, taxid:4097], or non-human, non-mouse expressed sequence tags [est_others]. All of the indicated alignments represent *N. tabacum* GenBank entries.

BLAST alignments of the cloned Myb27 tobacco flanking sequences (NtL, NtR-B1 and NtR-B2) to GenBank (full nucleotide collection and the tobacco Genomic Survey Sequence, taxid 4097) produced no perfect matches but did identify multiple GenBank entries with highly similar sequences (Fig. 5). The matching GenBank sequences were all from *N. tabacum* and clustered into three discontinuous sets of related sequence entries. No identified GenBank entry contained tobacco sequence that was contiguous across any two of the three alignment clusters (NtL, NtR-B1 and NtR-B2, Fig. 5). Attempts to PCR amplify across the NtL::NtR-B1, NtL::NtR-B2, and NtR-B1::NtR-B2 sequence boundaries was successful only using Myb27 DNA, with DNA from untransformed tobacco failing to produce the predicted DNA fragments (Additional file - Fig. S3-A). Amplification using PCR primer sets internal to each of the three tobacco DNA segments (e.g sets 6 and 7, Fig. 1) all produced a similar pattern of bands using either Myb27 or untransformed SR1 tobacco DNA as template (Additional file - Fig. S3-B). The Myb27 and SR1 set 1, 6 and 7 (Fig. 1) amplification products were directly sequenced, revealing the presence of numerous size and/or nucleotide polymorphisms (data not shown). It appears that all three plant DNA segments found adjacent to the single TDNA insert in the Myb27 transgenic line are imperfectly repeated within the tobacco genome.

In summary, the single TDNA insert in the Myb27 line, although lacking ∼650 bp at the Bar gene end of the original TDNA, was not internally rearranged during insertion into the tobacco genome. The Myb27 TDNA locus is flanked by three non-contiguous tobacco DNA segments that are likely to have been brought into proximity during TDNA integration into the tobacco genome.

### Silencing of the 35S::*AtMYB90* transgene is associated with accumulation of siRNAs molecules, primarily 21 nt long and homologous to both strands of the *AtMYB90* 3′ region

Total leaf RNA was isolated from several plant samples: SR1 (wild type tobacco); 27Hmo (silenced leaves, from two independent samples: 27Hmo-O1 & 27Hmo-LL6); 27Hmi (unsilenced, purple leaves); 237Hmo (unsilenced, purple leaves); 27Hmi–237Hmi (silenced leaves); and 27Hmi-SysSil (silenced leaves distal to the *Agrobactrium* infiltration site). These RNA samples were size fractionated (<40 nt), ligated to adaptors, converted to cDNA, and gel purified for deep-sequencing using the SOLiD™ platform [41]. The resulting data sets (colorspace data files converted to FASTA format), were aligned to a database of mature plant microRNA (miRNA) sequences (PMRD, 6220 unique entries on August 29, 2011) [42]. Sequence data from all plant samples produced multiple alignments to several highly conserved plant miRNAs (stringency set to 100% homology with the mature miRNA sequence), indicating that the sequenced samples all contained RNA of the appropriate size and had been effectively converted to PCR-amplified cDNA for sequencing (example miRNAs read numbers are listed in Table 1).

**Table 1:** Plant mature miRNA reads in small RNA sequence data sets.

miRNA*	27H-O1	27Hmo-LL6	27Hmi–237Hmi	27Hmi-SysSil	27Hmi	237Hmo	SR1
aqc-miR156a	13644	3101	1563	7745	316	437	739
csi-miR166	29814	3640	17152	10408	2391	1555	3368
vvi-miR396b	102406	6182	7361	21032	1034	1046	3158
nta-miR828	811	124	670	1252	25	11	0
Total Reads (×106)	22.36	5.05	5.10	9.7	12.5	17.16	20.48

*aqc = *Aquilegia caerulea*, csi = *Citrus sinensis*, vvi = *Vitis vinifera*, nta = *Nicotiana tabacum*.

**Figure 6:**
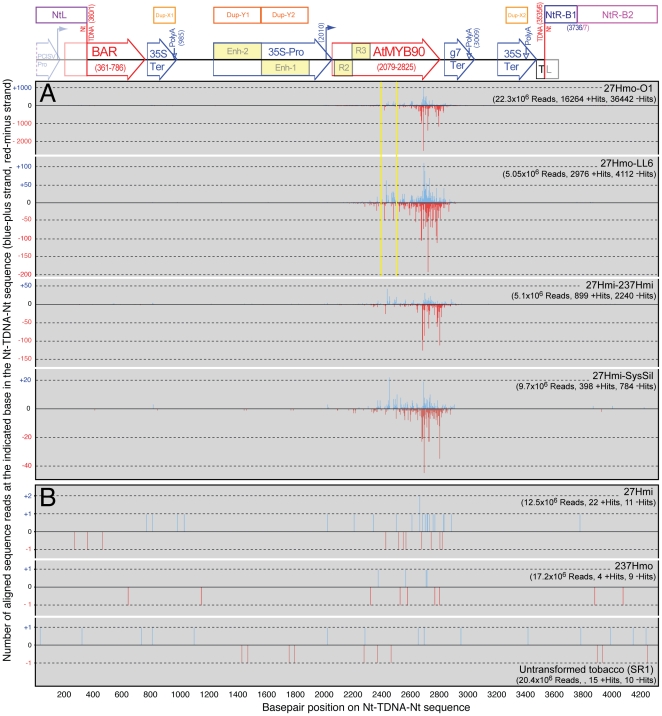
Small RNA sequence alignments mapped to the Myb27 TDNA and flanking tobacco sequence. Short RNA sequence reads (SOLiD platform) from plant samples displaying a silenced (**A**) or purple (**B**) phenotype were BLAST aligned to the Myb27 Nt-TDNA-Nt sequence. A structural map of the alignment target sequence is shown at the top (see Fig. 1 for labels). The TDNA contains two areas of duplicated sequence: Dup-X (X1 = 35S-PolyA: 841–1000; X2 = 3280–3439) and Dup-Y (duplicated 35S promoter enhancer region: Y1 = 1264–1590; Y2 = 1592–1918). It is impossible to determine which repeat represents the actual origin of any of the small number of matching siRNAs and the point of homology has been marked at both copies. For each RNA sample the total number of unfiltered sequence reads, and the number of TDNA alignments, is indicated. Yellow vertical lines indicate the segment of *AtMYB90* displayed in Fig. 9 (2367–2532).

The individual smRNA sequence data sets (ranging from 5×10^6^ to 22×10^6^ individual, unfiltered reads per RNA sample, see Table 1) were BLAST aligned and mapped to the Myb27 TDNA sequence (including the adjacent tobacco segments: NtL, NtR-B1 and NtRB2). Only reads with 100% homology and between 19 and 25 nucleotides in length were included in the graphic representation of the mapped reads (Fig. 6). The smRNA sequences from silenced material all contained large numbers of reads with homology to the Myb27 TDNA locus (27Hmo-O1 = 52703, 27Hmo-LL6 = 7087, 27Hmi–237Hmi = 3139, and 27Hmi-SysSil = 1182). The majority of these sequence reads (>98.5%) mapped to the *AtMYB90* transcribed region (Fig. 6 A). Samples from phenotypically non-silenced (purple) transgenic plants contained very few sequence reads that mapped to the Myb27 TDNA (27Hmi, 33 reads and 237Hmo, 13 reads, Fig. 6 B).

In summary, the transgene silenced phenotype (reduced anthocyanin pigmentation) was consistently associated with the presence in leaf tissues of abundant siRNA with homology to both strands of the 3′ end of the *ATMYB90* transcribed region.

### Silenced tissues display distinctive profiles for siRNAs mapped to the *AtMyb90* 3′ sequence

**Figure 7:**
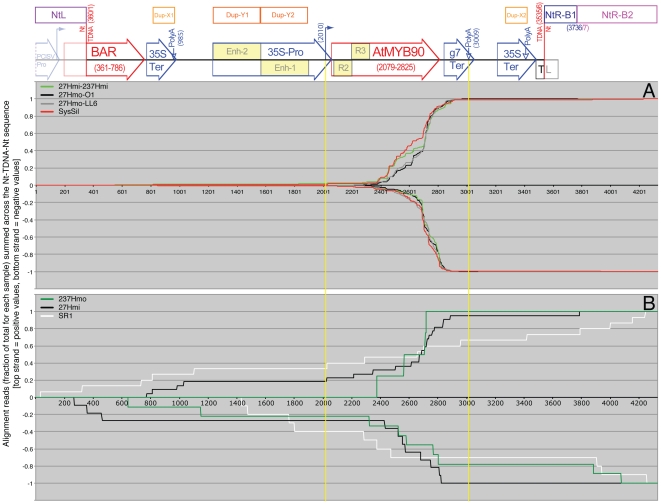
Normalized siRNA profiles summed across the Nt-TDNA-Nt from Myb27. The number of aligned sequence reads from each sample (Fig. 6) were divided by the total number of matching reads for the corresponding sample (plus [above 0 line] and minus [below 0 line] strands were treated separately) and the resulting fractions summed across the Nt-TDNA-Nt sequence (from 0 at position 1 to 1.0 at position 4329). Profiles from silenced (A) or non-silenced (B) phenotypes are aligned to a structural map of the Nt-TDNA-Nt target sequence (diagrammed at the top, see Fig. 1 for labels). See the legend for Fig. 6 regarding how regions of duplicated sequence were addressed. The boundaries of the *AtMYB90* transcript are indicated by yellow vertical lines.

In order to directly compare siRNA profiles from different samples, the read alignment values plotted in Fig. 6 (19 nt–25 nt, 100% homology) were normalized to the total number of aligned sequence reads for each data set (positive strand and negative strand data were treated separately) and the resulting fractions summed (0.0 at the left to 1.0 at the right) across the entire Myb27 TDNA locus (including flanking tobacco genomic sequence [Nt-TDNA-Nt], Fig. 6 A & B). The resulting profiles provide a visual representation of the distribution of aligned siRNA reads and confirmed a tight clustering of silenced sample siRNAs to the *AtMYB90* transcribed region. The two independent 27Hmo replicates (O-1 and LL6) showed very similar summation patterns. The 27Hmo profiles, however, differed from the those generated using 27Hmi-SysSil and 27Hmi–237Hmi sequence reads (see sense strand profiles, Fig. 7 A).

The limited number of alignments identified in control, untransformed tobacco RNA (SR1 - 23 reads) were randomly distributed across the Myb27 TDNA target sequence, Nt-TDNA-Nt (Fig. 7 B). The NtL, NtR-B1 and NtR-B2 segments of the Nt-TDNA-NT alignment target, representing the only portions of that sequence that are actually present within untransformed tobacco, did not display a significantly different density of smRNA alignments when compared to the introduced TDNA sequence. A similar lack of siRNA sequence reads matching the flanking tobacco DNA was seen with all of the other RNA samples analyzed, including those that contain abundant *AtMYB90*-derived siRNAs. The NtL, NtR-B1 and NtR-B2 tobacco sequences do not appear to be intrinsic sources of smRNAs, at least within the leaves of plants propagated under our normal growth conditions.

The few TDNA-derived sequence reads identified within purple, unsilenced, transgenic plant samples (27Hmi and 237Hmo) were similar in number to those seen in the SR1 sample (SR1 - 23 reads, 27Hmi - 33 reads and 237Hmo - 13 reads). However, when compared to the remainder of the Nt-TDNA-Nt sequence, mapped 27Hmi siRNA reads were enriched within the *AtMYB90* transcribed region. A chi-square test using siRNA alignment densities (aligned reads per kb of sequence) from 27Hmi, 27Hmo and 237Hmo samples, paired with the randomly distributed SR1 sample, indicated that sequence reads from both 27Hmo and 27Hmi samples clustered to the *AtMYB90* transcribed region (sequence range 2010–3009), relative to the remaining TDNA sequence (including flanking tobacco DNA). While not as obvious as the *AtMYB90* clustering seen in the silenced 27Hmo (p<0.0001), clustering of the 27Hmi reads to the AtMYB90 transcribed region was still statistically significant (p = 0.0019). Although alignments from the 237Hmo sample appear to weakly cluster within the *AtMyb90* region, the number of aligned sequence reads was insufficient for statistical confirmation (p = 0.0659).

**Figure 8:**
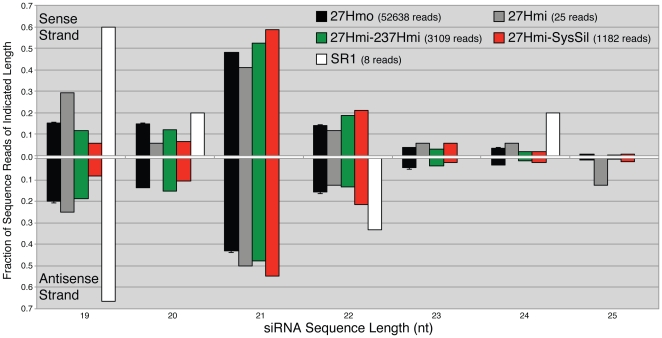
SmRNA size profiles for siRNAs homologous to the *AtMYB90* transcribed region. Each bar represents the number of sense (top) or antisense (bottom) siRNAs of the indicated size divided by the total number of siRNA sequence reads aligning to the *AtMYB90* transcribed region (2010–3009 bp of Fig. 1, sense and antisense data were independently normalized). Sequence from replicate samples of the 27Hmo treatment (O1 and LL6) were used to generate the 27Hmo values shown (standard error indicated, n = 2).

**Figure 9:**
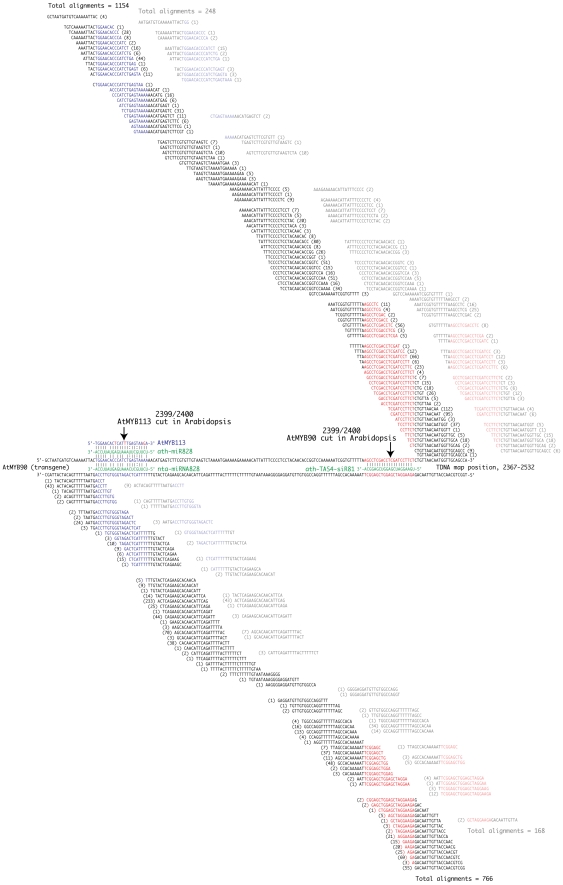
siRNA sequence alignments to Nt-TDNA-Nt segment 2367–2532. siRNA sequence reads from 27Hmo-O1 and 27Hmo-LL6 (LL6 text is faded and offset to the right ∼20 nt) siRNA sequence reads directly aligned to the 2367 to 2532 segment of the Myb27 Nt-TDNA-Nt. The number of sequence reads for each alignment is shown in parenthesis next to the corresponding smRNA sequence. This sequence segment was chosen as it contains potential smRNA recognition sites identified in *Arabidopsis*. Homology to ath-miR828 (blue) and ath-*TAS4*-siR81 (red) are indicated. ath-*TAS4*-siRNA81 associated cleavage of *AtMYB90* and miRNA828 directed cleavage of the closely related *AtMYB113* mRNA (arrows) have been detected in *Arabidopsis* [43].

For all four silenced plant samples, siRNAs homologous to the *AtMYB90* transcribed region were primarily 21 nt long (Fig. 8), with this length representing approximately half of all matching reads (analysis was limited to sequence reads 19 nt–25 nt long with 100% homology). The fraction of aligned siRNAs reads at each length (19 nt–25 nt) was similar for both sense and anti-sense strands. Despite a limited number of aligned reads (25 reads with *AtMYB90* homology), the unsilenced 27Hmi sample displayed a size profile similar to that observed in the silenced samples, with 21 nt siRNAs comprising roughly half the total number of reads (Fig. 8). The profile seen with the small number (8) of tobacco control *AtMYB90*-aligned reads (SR1) is skewed towards shorter 19 nt lengths consistent with the SR1 alignments representing a background of random sequence reads. A short representative segment of the *AtMYB90* coding region (2659–2796), displaying matching 21 nt siRNA sequences (from the 27Hmo-O1 and 27Hmo-LL6 samples) directly aligned with the transgene sequence, is shown in Fig. 9. This segment of the TDNA was chosen for more detailed analysis due to the presence of two potential small RNA binding sites in *Arabidopsis* [43]. The siRNA aligned to this region did not indicate any unique pattern of siRNA molecules with homology to either site.

In summary, the distribution of siRNAs targeted to the *AtMyb90* transgene was reproduced in two biological replicates of silenced 27Hmo leaf material. The siRNA profiles for 27SysSil and 27Hmi–237Hmi samples were similar, but not identical, to each other and to the 27Hmo profile. Unsilenced 27Hmi leaf samples contained a limited number of sequence reads homologous to the Myb27 Nt-TDNA-Nt sequence (∼0.01% of the number of reads in 27Hmo silenced samples) that cluster to the same segment of the *AtMyb90* transcribed region targeted in the silenced samples.

## Discussion

### 35S::*AtMYB90* - silencing and gene dosage

Transgene silencing has always played an important, if poorly understood, role in both research and commercial work involving plant genetic engineering [1], [2], [3], [4], [44], [45], [46]. Despite this impact, the actual molecular factors that contribute to initiating silencing of introduced genes remain obscure. Duplication, rearrangement and/or read-through transcription of introduced DNA has been found to be strongly associated with transgene silencing and likely reflect an unintended production of dsRNA. Silencing of single copy transgenes also occurs in a subset of plant transformation events and has occasionally been found to be triggered by the doubling of transgene copy number associated with plants made homozygous for the introduced DNA [24], [25], [26], [27], [28], [29], [30], [31], [32]. These transgenic lines present an opportunity to examine initiation of silencing in a controlled and otherwise isogenic environment.

Of the published reports describing homozygosity-linked transgene silencing, most have involved transformed tobacco, have made use of the highly active CaMV-35S viral promoter, and have been described as involving PTGS. It has been generally assumed that the doubling of transgene transcription in homozygous plants resulted in the mRNA levels (or the levels of altered versions of each transcript) exceeding a threshold and triggering transgene silencing [1], [5], [23], [24], [32], [44], [47]. However, many of the homozygosity-associated silenced lines displayed silencing in only a subset of homozygous plants (Nitrate reductase [24], [31], [37], [47], *rolB* [48] firefly luciferase [26], chitinase [25]), indicating a stochastic component to the trigger and complicating effective molecular analysis. Our visually screenable Myb27 line is reproducibly silenced in all homozygotes, consistently reverts to a non-silenced state in hemizygous plants, and does not contain an identical endogenous tobacco gene that would complicate analysis of transgene-associated smRNA [33]. As a first step in examining the initiation of silencing in 27Hmo leaves we have examined the population and characteristics of smRNAs targeting the *AtMYB90* transgene in silenced (27Hmo, 27Hmi–237Hmi, 27Hmi-SysSil) versus non-silenced (27Hmi, 237Hmo) plants.

### Transgene and TDNA structure

The genetics of the Myb27 dominant purple phenotype and structure of Myb27 TDNA insertion are consistent with the presence of a single 35S::*AtMyb90* transgene that is co-linear with that occurring in the original TDNA construct. This leaves open the question as to what characteristic(s) of the Myb27 transgene are responsible for promoting silencing in 27Hmo plants. The structure of the cloned Myb27 insertion indicates that a deletion of the TDNA right border region, and some reorganization of the flanking tobacco DNA, occurred during integration. However, there is no indication of any duplication and/or inversion of the *AtMYB90* coding region. In support of there being a single functional copy of the *AtMYB90* transgene in Myb27, a spontaneous single-base nonsense mutation within the *AtMYB90* coding region converted the phenotypically dominant Myb27 transgene locus (indicated by anthocyanin accumulation) into a dominant-negative marker that interfered with normal tobacco flower pigment production [33].

The observed juxtaposition of what appear to be unlinked and repetitive tobacco sequence segments adjacent to the Myb27 TDNA insert indicate that alterations to the tobacco genomic DNA arose during integration. However, there is no indication that these flanking sequences are highly transcribed (e.g. few GenBank est database hits, Fig. 5), nor were they the target of natural smRNAs (all of the sequence data sets contained very few siRNA reads homologous to the flanking tobacco DNA, Fig. 6). In addition, tight clustering of the majority of mapped siRNAs to the 3′ portion of the Myb27 *AtMyb90* coding region make it unlikely that the siRNA is the result of transitive spread of silencing from flanking tobacco DNA. It appears that phenotypically detectable transgene silencing in these purple plant lines is initiated by an alteration to some aspect of the *AtMYB90* mRNA associated with the doubled transgene copy number present in 27Hmo or 27Hmi–237Hmi plants.

### *AtMYB90* transgene silencing

The observed reduction in 27Hmo steady-state mRNA (Fig. 3), altered northern blot (Fig. 3) and presence of transgene-derived siRNA (Fig. 6), are all consistent with a PTGS mechanism for the observed silencing. In all silenced samples sequenced, transgene siRNA homologous to both strands of the *AtMYB90* transcribed region were identified (Fig. 6), suggesting the participation of an RdRP in siRNA production. The transgene-derived siRNA is primarily 21 nt long (Fig. 7), implicating the involvement of *DICER-LIKE 1* (*DCL1*) and/or *DICER-LIKE 4* (*DCL4*), in the processing of siRNA precursor(s). The sequenced samples showed a relatively low abundance of 24 nt siRNAs, the product of *DICER-LIKE 3* (*DCL3*) activity, making it unlikely that the observed 27Hmo transgene silencing results from DNA methylation and transcriptional silencing (a conclusion also reported for systemic silencing in grafted scion tissues [49], [50]).

It is interesting that the siRNA profiles seen with the 27Hmi–237Hmi and SysSil samples (see plus strand patterns, Fig. 7) do not directly parallel those from the biologically replicate 27Hmo samples (O1 and LL6), possibly reflecting alternative pathways for siRNA production and/or processing in these samples.

### Triggering transgene silencing in Myb27 plants

The similarity in the pattern of silencing seen with systemically silenced 27Hmi plants and gene-dosage silenced 27Hmo plants (see 27Hmi-SysSil and 237Hmi-SysSil, Fig. 2) are consistent with a common cellular origin for silencing in these leaves. In systemically silenced plants a transgene-specific smRNA signal is transported from source cells to a distal tissue where it triggers silencing in at least a subset of cells [51], [52], [53], [54], [55], [56]. Silencing at the distal site appears to be the result of smRNA recognition within targeted cells, coupled with short distance spreading to, and amplification within, adjacent cells [51], [56], [57]. It is possible that 27Hmo silencing also initiates within a subset of leaf cells (likely located in or near leaf veins), followed by spreading and siRNA amplification. If the *AtMYB90* mRNA is subject to some form of low-level cleavage and/or modification unique to specific cell types, elevated transcription could produce sufficient aberrant RNA within those cells, triggering transgene siRNA production, maintenance and spread.

One possible source of aberrant *AtMYB90* RNA in the Myb27 line may involve the homology between *AtMYB90* mRNA and two *Arabidopsis* smRNAs; one, a highly conserved miRNA, ath-miR828, and the second; a trans-acting siRNA (tasiRNA), ath-*TAS4*-siRNA81. Work done in *Arabidopsis* identified the tasiRNA binding site in *AtMYB90* mRNA as a target of *in vivo* cleavage [43]. In addition, there is a functional linkage between these two smRNAs in that miR828-guided cleavage of *TAS4* transcripts is required for production of ath-*TAS4*-siRNA81 molecules in *Arabidopsis* [43]. If a homolog to one or both of these smRNAs is active in tobacco it could be responsible for cleavage of the 35S::*AtMYB90* transcript in an area near the 5′ boundary of the of siRNA cluster (yellow lines, Fig. 6). A close examination of 27Hmo 21 nt (Fig. 9) and 22 nt (not shown) siRNA alignments within the miRNA828 to TAS4-siRNA81 segment of *AtMYB90* does not indicate an unusual number or pattern of siRNA molecules with homology to either site , nor is there any indication of siRNA phasing that might be expected after tasiRNA-like processing of double-stranded *AtMYB90* RNA that had been cut at the miR828 site. It is possible that any smRNA-based evidence of specific miR828 or *TAS4*-siRNA81 cleavage/siRNA-phasing of *AtMYB90* mRNA, had been diluted by subsequent siRNA amplification and the spread of silencing to adjacent cells. Curiously, all the silenced samples show nta-miR828 read abundances that are dramatically elevated relative to unsilenced transgenic samples (Table 1). The possibility of tobacco-miR828 guided cleavage of the *AtMYB90* message in Myb27 is complicated by the relatively low complementarity with the mRNA target sequence (Fig. 9). However, it is becoming apparent that full base pairing between smRNA and target sequences is not required for functional interaction [58]. The possible involvement of smRNAs similar to miR828 and/or *TAS4*-siRNA81 in Myb27 silencing will require further investigation. For example, sequencing of smRNAs could be focused on tissues within and near leaf veins in young silenced (27Hmo) or unsilenced (27Hmi) leaves. Also, *AtMyb90* mRNA from those tissues could be examined for miR828 or *TAS4*-siRNA81 directed cleavage of the *AtMyb90* transcript using rapid amplification of cDNA Ends (RACE).

## Materials and Methods

### Genes and constructs

The pZP35SMYB construct (diagrammed in Fig. 1) has been described previously [21], [33]. The Myb hairpin construct, pKOihpMyb, is related to the pKOihpFLUC plasmid [21], with the pKOihpFLUC luciferase coding segments replaced by the segment of *AtMYB90* coding region indicated in Fig. 1 (red bar), in a tail-to-tail orientation.

### qRTPCR

Protocols for PCR and qRTPCR are as described previously [33]. Relative RNA levels were calculated using 3× technical replicates for each of 2–3 biological replicates. Except when indicated, middle leaves (5^th^–9th true leaves) were used for mRNA analysis.

### Cloning and sequencing of tobacco-TDNA junctions , cDNAs and RACE products

The TDNA borders plus adjacent plant sequences were cloned from 27Hmo total DNA using thermal asymmetric interlaced polymerase chain reaction (TAIL-PCR [40]) and adaptor-primed PCR (GenomeWalker™) according to the manufacturer's suggested protocols.

*AtMYB90* mRNA was converted to cDNA as described previously [33], and end fragments PCR amplified using an Expand Long Range PCR kit from Roche Applied Science. PCR fragments from cDNA were directly sequenced using an ABI 3130xI Genetic Analyser and Big Dye® Terminator kit v3.1 as per the manufacturer's instructions.

The transcription start and polyA addition sites were determined using an invitrogen™ GeneRacer™ kit, following manufactures instructions designed to specifically amplify capped 5′ mRNA ends and insure the correct 5′ transcription start site of mature 35S::*AtMYB90* transcripts.

### SOLiD™ sequencing of small RNA

Small RNA sequence data from tobacco samples were obtained using the Applied Biosystems SOLiD™ v3.0 or v4.0 platforms. For all samples, 100 µg of total RNA (from leaves in which the central vein had been excised) was enriched for small RNA (≤40 nt) using the Ambion® FlashPAGE™ system. Enriched smRNA was cleaned and concentrated using Ambion® spin columns and all concentrations were determined using the Invitrogen® Qubit® fluorometer and the Quant-iT™ RNA assay kit (Molecular Probes® detection technologies). The Quant-iT™ RNA assay kit was performed according to manufacturers protocols with 3 µl of the enriched RNA sample. Based on measured concentrations, a total of 50 ng of each of the smRNA enriched samples was used to construct smRNA libraries with the Ambion® Small RNA Expression Kit (SREK). SREK protocols have been optimized to generate fragments for sequencing representing all small, non-coding RNAs in a sample that range in size from ∼18–40 nt. Briefly, an adapter (Adapter mix A) recognizing the 5′-monophosphate was ligated to each smRNA sample to ensure sequencing of all templates from the 5′ end. Following adapter ligation, cDNA was synthesized by reverse transcription with subsequent RNAseH treatment to remove any RNA/cDNA duplexes and any free adapters or adapter by-products. After conversion into cDNA each library was amplified using one of 10 primer sets included with the SREK kit in order to both append the terminal sequences necessary for SOLiD™ sequencing and to incorporate unique barcodes onto each library. The primer sets used for amplification are identical except for a 6 bp barcode (sequence tag) incorporated into the 3′ primer. Amplification was followed by polyacrylamide gel electrophoresis (PAGE) purification on 6% non-denaturing gels and size selection of 100–150 bp products that were used for attachment to sequencing beads and emulsion PCR (ePCR). All ePCR and bead deposition techniques were done following Applied Biosystems standard protocols. Specifically, a total of 60 pg from each of the purified smRNA libraries was used in two different ePCR reactions. After bead clean up, enrichment of p2 beads, and 3′modification according to standard protocols (Applied Biosystems V3.0 manual, Applied Biosystems) a total of 70 million beads (35 million from each ePCR) containing the 6 barcoded libraries were deposited into two quadrants of a SOLiD™ sequencing slide. Sequencing was done following a bar-code sequencing run to identify the six different libraries and as suggested for eukaryotic miRNA library sequencing 35 bp (V3.0), or 50 bp (V4.0) reads were collected using the appropriate SOLiD™ fragment library sequencing kit.

The resulting data were converted to FASTA format and analyzed using a combination of software packages, including NextGENe® (www.Softgenetics.com), GeneSifter™ from Geospiza, Inc (www.geospiza.com) and BioEdit v7.9.1 (www.mbio.ncsu.edu/bioedit/bioedit.html). Local BLAST alignments used BioEdit v7.9.1 and a 4029 bp target sequence representing the flanking tobacco DNA and TDNA present in the Myb27 line (represented in Fig. 1).

### Statistical analysis of siRNA homology to Myb27 TDNA

Counts of siRNA reads per thousand base pairs present in the *AtMyb90* transcribed region and remaining TDNA were determined separately to create a series of cross-tabulation tables. For each treatment chi-square statistics were calculated to assess for an association between variables using the count response. The chi-square tests were performed using the FREQ procedure in SAS version 9.2.

### miRNA bioinformatics

The SOLiD™ sequence data sets were matched to a miRNA database derived from the plant microRNA database <http://bioinformatics.cau.edu.cn/PMRD/?> [42] using custom Python scripts. The searched miRNA collection was limited to unique miRNA sequences from the PMRD database (6,220 entries). Sequence reads with the longest contiguous character-by-character match (100% homology) to the full length of a mature miRNA sequence entry were counted.

### Blot hybridization

Blot production and hybridizations were performed as described previously [59].

### Callus culture of leaf tissue

Callus growth was induced on surface sterilized leaf explants placed on MS-agar media [60] supplemented with plant hormones (MS Salt; B5 Vitamins; Sucrose 2% [w/v]; indol- 3-acetic acid (0.5 mg/mL); benzlaminopurine (0.5 mg/mL). Culture plates were incubated at 28°C with an 18/6-hour light/dark cycle.

## Supporting Information

Figure S1 ***AtMyb90*** **transcribed region.** The nucleotide position numbers are those from the cloned Myb27 TDNA, including flanking tobacco sequences (Fig. 1). The transcribed region of the 35S::*AtMYB90* transgene is shown (blue text = R2 domain, red text = R3 domain). The area of sequence containing 95% of all the mapped siRNA sequence reads from silenced samples is boxed. The locations of ath-miR828 and ath-*TAS4*-siR81 homologies are underlined [43].
(TIF)

Figure S2 **Reversal of silencing in culture.** Leaf segments from green sectors of 27Hmo leaves were surface sterilized and placed on MS media with and without hormones. Callus resulting from induced cellular division did not display silencing of the 35S::*AtMYB90* transgene as indicted by the accumulation of high levels of anthocyanin pigments.
(TIFF)

Figure S3 **Myb27 Nt-TDNA-Nt PCR from genomic DNA.** PCR results from the indicated primer sets (Fig. 1 and Table 1) using total genomic DNA isolated from transgenic (**27Hmo**) and wild type (**SR1**) plants. Restriction enzyme digests (*Pst*I) of lambda bacteriophage DNA were used as size markers (sizes are indicated). The PCR product sizes predicted for each primer set are indicated in parentheses.
(TIF)

Table S1 **PCR primer sets.** The DNA sequence for PCR primer pair sets 1through 7 and for the *AtMYB(90)* qRTPCR primers are listed, including product size for each product (in base pairs).
(DOC)
