# Supplementary figures and images for: Transgene Silencing and Transgene-Derived siRNA Production in Tobacco Plants Homozygous for an Introduced AtMYB90 Construct
Source: PLoS One. 2012 Feb 17;7(2):e30141. doi: 10.1371/journal.pone.0030141 (PMC3281821; doi:10.1371/journal.pone.0030141)

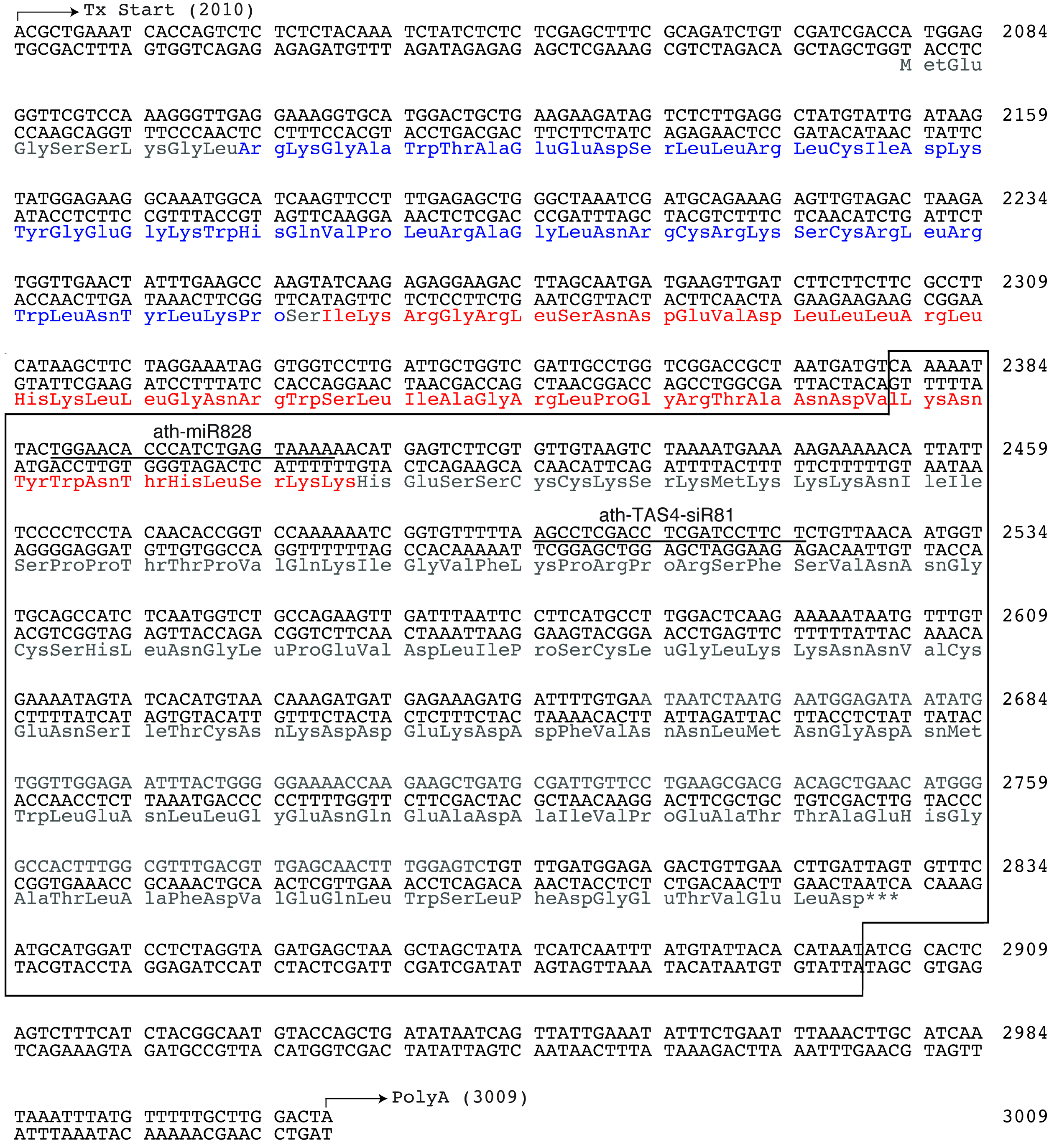

Supplement: Figure S1 — AtMyb90 transcribed region. The nucleotide position numbers are those from the cloned Myb27 TDNA, including flanking tobacco sequences (Fig. 1). The transcribed region of the 35S::AtMYB90 transgene is shown (blue text = R2 domain, red text = R3 domain). The area of sequence containing 95% of all the mapped siRNA sequence reads from silenced samples is boxed. The locations of ath-miR828 and ath-TAS4-siR81 homologies are underlined [43]. (TIF) [file pone.0030141.s001.tif]

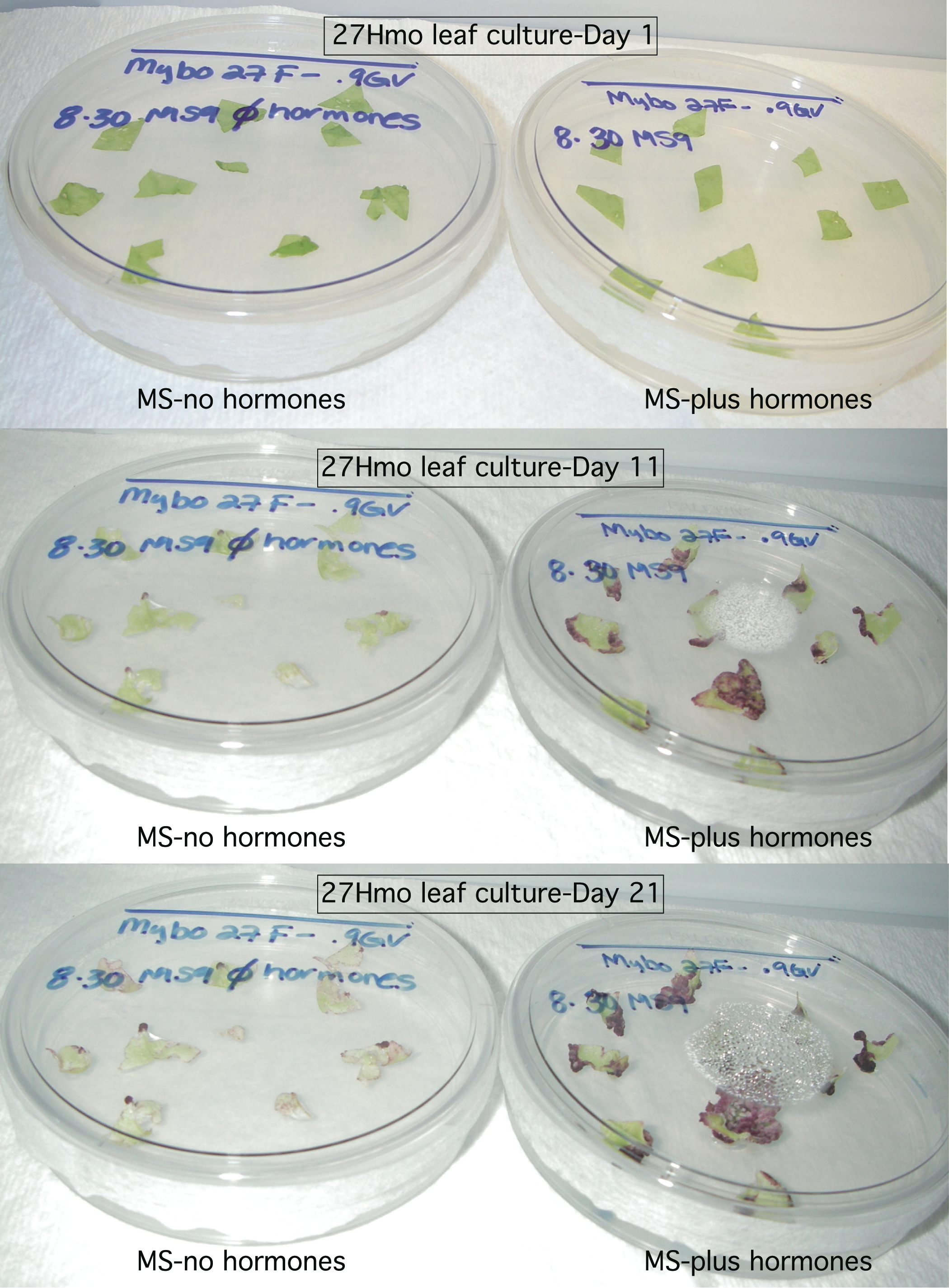

Supplement: Figure S2 — Reversal of silencing in culture. Leaf segments from green sectors of 27Hmo leaves were surface sterilized and placed on MS media with and without hormones. Callus resulting from induced cellular division did not display silencing of the 35S::AtMYB90 transgene as indicted by the accumulation of high levels of anthocyanin pigments. (TIFF) [file pone.0030141.s002.tif]

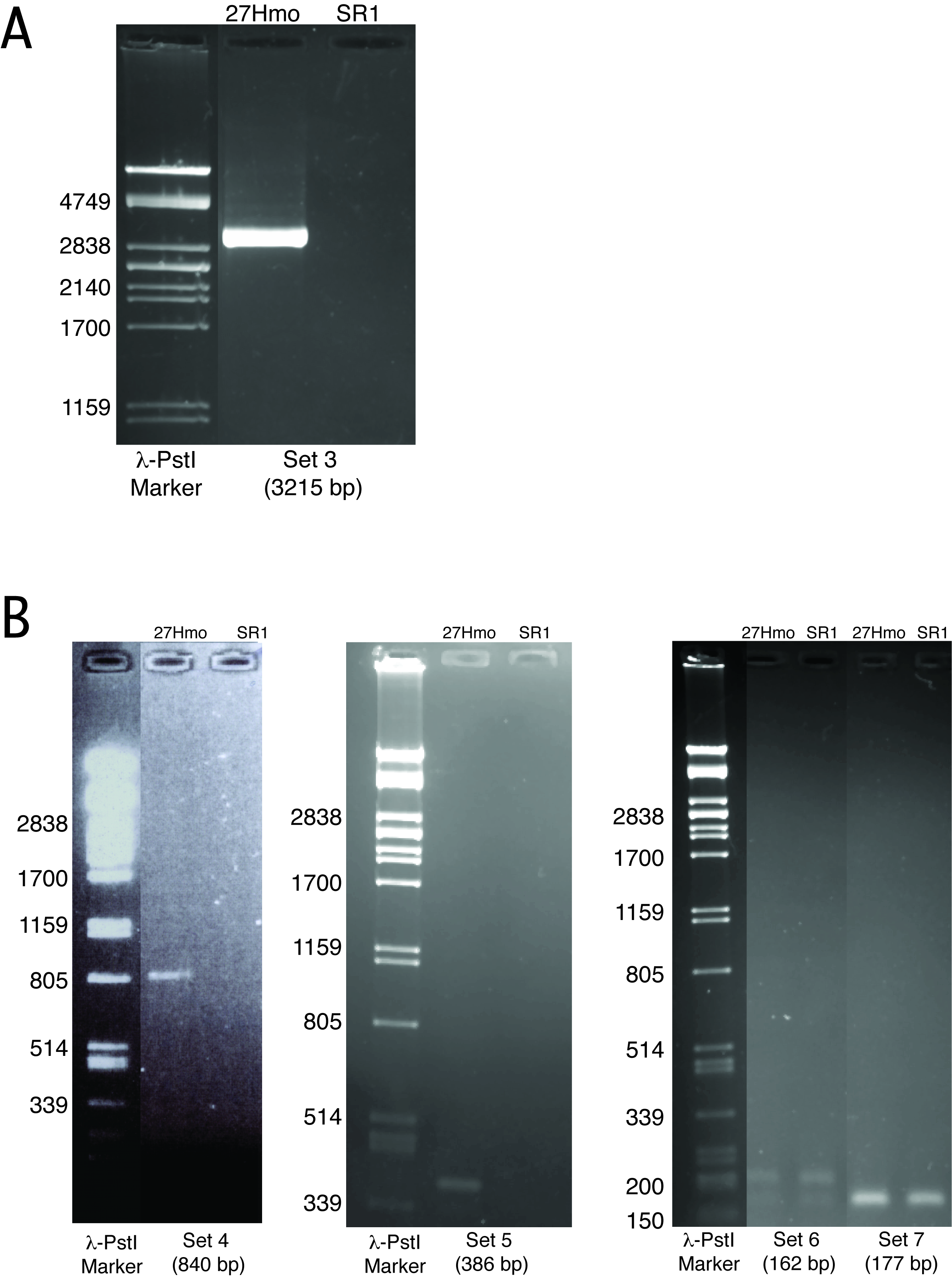

Supplement: Figure S3 — Myb27 Nt-TDNA-Nt PCR from genomic DNA. PCR results from the indicated primer sets (Fig. 1 and Table 1) using total genomic DNA isolated from transgenic (27Hmo) and wild type (SR1) plants. Restriction enzyme digests (PstI) of lambda bacteriophage DNA were used as size markers (sizes are indicated). The PCR product sizes predicted for each primer set are indicated in parentheses. (TIF) [file pone.0030141.s003.tif]
