# Supplementary material for: Transgene Silencing and Transgene-Derived siRNA Production in Tobacco Plants Homozygous for an Introduced AtMYB90 Construct
Source: PLoS One. 2012 Feb 17;7(2):e30141. doi: 10.1371/journal.pone.0030141 (PMC3281821; doi:10.1371/journal.pone.0030141)
Supplement: Table S1 — PCR primer sets. The DNA sequence for PCR primer pair sets 1through 7 and for the AtMYB(90) qRTPCR primers are listed, including product size for each product (in base pairs). (DOC) [file pone.0030141.s004.doc]

Supporting Information – Table S1: PCR primer sets

| Set | Forward (5’->3’) | Reverse (5’->3’) | Product (bp) | |
| --- | --- | --- | --- | --- |
| 1 | ATTGACCCTTTGACAAATCACG | AGCCCGATGACAGCGAC | 265 |  |
| 2 | GAACTGCCACCGGGCATT | AGCCCGATGACAGCGAC | 474 |  |
| 3 | CCTAGATCCTTGTCCTTGTCTATACTCCTCAGG | TGCTGAGCTTGGAAACAAATTGACG | 3215 |  |
| 4 | ACATAATATCGCACTCAGTCTTTCATC | ACCCTCAAACTATTTATGTTTTATATTTGATATTG | 840 |  |
| 5 | CCGGAAAGGCACACAATATC | AAACTAGCTTGCACTAATGTCATACTTATAATATTC | 386 |  |
| 6 | CCGGAAAGGCACACAATATC | CAATATCAAATATAAAACATAAATAGTTTGAGGGT | 162 |  |
| 7 | TAATAGAACTGAACGTGACTAACATTATTTATATGTC | GAATATTATAAGTATGACATTATTAGTGCATAGCTAGTTT | 177 |  |
| qRTPCR | GACTGCTGAAGAAGATAGTCTCTTG | GCCCAGCTCTCAAAGGAACTTGATG | 80 |  |
